# Long-term event-free survival with an embolised prosthetic valve leaflet in the thoracic aorta

**DOI:** 10.1186/1749-8090-3-34

**Published:** 2008-05-30

**Authors:** Mohan P Devbhandari, Edwin BC Woo, Timothy L Hooper

**Affiliations:** 1 Department of Cardiothoracic Surgery, South Manchester University Hospital, Manchester, UK

## Abstract

We report the case of a patient who underwent a redo surgery for a leaflet escape from a Bjork-Shiley tilting disc mitral prosthesis inserted 18 years previously. The escaped disc remained lodged in the thoracic aorta without any complication. She ultimately died of terminal heart failure 13 years after the second operation. We believe this to be the longest survival with a dislodged leaflet from a mechanical valve. Removal of dislodged disc is recommended in literature but there may be a place for watchful observation in exceptional cases with no haemodynamic compromise.

## Introduction

Leaflet escape from a prosthetic valve is a rare but life-threatening event. Treatment is by emergency replacement of the prosthesis and retrieval of the escaped leaflet. Reported literatures till date suggest that failure to retrieve the embolised leaflet will lead to arterial or hemodynamic complications that may have dire consequences for the patient [1].

## Case report

A 52 year old lady underwent mitral valve replacement with a 23 mm Björk-Shiley prosthesis18 years previously for rheumatic mitral stenosis. Latterly she had suffered with refractory supraventricular tachyarrhythmias and a transeptal AV node ablation was attempted. Unfortunately she became acutely unwell during the procedure, with cardiogenic shock and pulmonary oedema, which proved to be due to escape of the disc-occluder from the valve prosthesis during the cardiological maneuver. She underwent emergency surgery to replace the damaged valve with a bileaflet device, but the escaped disc could not be found. It was later localized by trans-esophageal echocardiography to the descending thoracic aorta, with its long axis parallel to the long axis of aorta, thus not interrupting the flow.

Postoperatively, she had a protracted intensive care stay with multi-system failure, and was not considered to be well enough to contemplate removal of the embolised leaflet. After discussion among the treating clinicians and family members a decision to "wait and watch" was adopted, with the question of further surgery being postponed until the patient's condition improved. She was eventually discharged from hospital after two months. By that time it was established that the patient did not want to pursue further intervention to remove the disc. She had been attending annual reviews for thirteen years and has had no problems referable to the embolised disc. She ultimately died of terminal heart failure. Post mortem examination was declined by the relatives.

## Discussion

Leaflet escape from a prosthetic valve has been reported following both mitral and aortic valve replacement surgery at variable intervals of time ranging from days to several years after the date of operation [2]. The causes for leaflet escape have been ascribed mainly to pivot system fracture [2,3] or disc fracture [4] though rarely it can follow interventional cardiological maneuvers as in our case.

The usual mode of presentation is with acute severe shortness of breath, often after a period of activity. Clinically, the picture is of acute left ventricular failure and pulmonary edema with cardiogenic shock, due to severe valvular incompetence [1-4]. Possible differential diagnosis that needs to be ruled out are myocardial infarction, para prosthetic valvular leak, malignant arrhythmia and pulmonary embolism.

Echocardiography is not always diagnostic of the leaflet escape and may be interpreted as showing obstructed closure of the prosthetic valve or a paravalvular leak. The picture can be confusing and misinterpreted as showing valve thrombosis resulting in anticoagulant therapy causing delay in life-saving surgery and death of patient [5].

Timely diagnosis and emergency surgical replacement of the damaged prosthetic valve is indicated. Delay in diagnosis or treatment may prove to be detrimental [2,5]. It is sometimes difficult to locate the missing leaflet which may have embolised more distally in the aorta [3,5] or iliac artery [1]. Plain radiographs often fail to visualize the disc as they are not sufficiently radio opaque. Ultrasound and CT scan are more accurate at localizing the dislodged leaflet [6,7] in most reported cases. Fluoroscopy [5] has also been used in some cases to localize the leaflet.

In the reported literature, it has been considered mandatory to retrieve the embolised disc at the same time or shortly after valve replacement [1]. Rarely the leaflets eluded all attempts at localization and were discovered only at autopsy [5].

There are only few reports of patient achieving long term survival, without complications, with a mechanical valve leaflet lodged in thoracic aorta. This we believe is the case with the longest survival. Previous authors have emphasized that it is mandatory to remove the foreign body due to risk of thrombosis, migration, erosion and infection at the site of lodgment. The critical condition of our patient at presentation along with absence of hemodynamic obstruction prompted us to follow a wait and watch policy against this recommendation, which proved a successful strategy.

## Conclusion

Following emergency surgery for prosthetic valve leaflet escape, there may be a place for watchful observation of the escaped disc if it is not causing haemodynamic compromise and the patient's condition or wishes prevents surgical removal.

## Consent

The patient has been deceased for several years and the relatives were not contactable at this time.  Please contact the editor for further details.

## Competing interests

The authors declare that they have no competing interests.

## Authors' contributions

MD designed the paper and prepared the manuscript, EW revised the manuscript, TH performed the operation and revised the contents of the paper. All authors read and approved the final manuscript.
